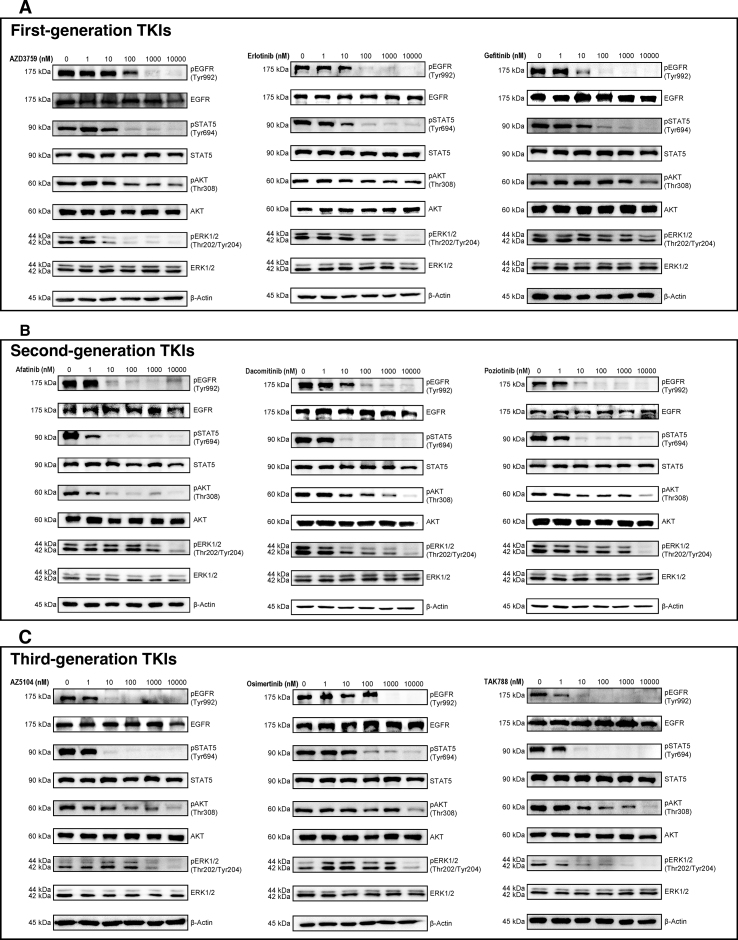# A novel acquired *EGFR-SEPT14* fusion confers differential drug resistance to EGFR inhibitors in lung adenocarcinoma

**DOI:** 10.1016/j.gendis.2023.02.019

**Published:** 2023-04-24

**Authors:** Weidong Wang, Wang Lv, Hui Wang, Yang Xu, Junrong Yan, Han-Ming Shen, Liqun Shan, Jian Hu

**Affiliations:** a Department of Thoracic Surgery, The First Affiliated Hospital, School of Medicine, Zhejiang University, Hangzhou, Zhejiang 310003, China; b Department of Thoracic Surgery, The First People's Hospital of Wenling, Wenling, Zhejiang 317599, China; c Medical Department, Nanjing Geneseeq Technology Inc, Nanjing, Jiangsu 210000, China; d Department of Physiology, Yong Loo Lin School of Medicine, National University of Singapore, Singapore 119077, Singapore; e Faculty of Health Sciences, University of Macau, Macau SAR 519000, China; f Department of Thoracic Surgery, Sun Yat-sen University Cancer Center, Guangzhou, Guangdong 510060, China; g Sun Yat-sen University Cancer Center, State Key Laboratory of Oncology in South China, Collaborative Innovation Center for Cancer Medicine, Guangzhou, Guangdong 510060, China

Around 10%–30% of non-small cell lung cancer (NSCLC) patients harbored epidermal growth factor receptor (*EGFR*) mutations, with L858R and exon-19 deletions (19-Del) accounting for 90% of cases. EGFR tyrosine kinase inhibitors (TKIs) showed significant efficacy against common *EGFR* mutations. However, the therapeutic relevance of uncommon *EGFR* mutations remained insufficiently investigated. *EGFR* fusions are extremely rare (0.05%–0.13%) in NSCLC, and Konduri group reported only 5 EGFR fusions from 10,097 patients.^1^^,^^2^ Additional *EGFR* fusions were reported in NSCLC,^3^^,^^4^ all of which were oncogenic drivers and sensitive to EGFR TKIs. Herein, we reported an NSCLC patient with leptomeningeal metastasis (LM) who acquired a novel *EGFR*-*SEPT14* fusion during TKI resistance and showed promising responses to certain EGFR TKIs and intrathecal pemetrexed (IP).

A 51-year-old male was diagnosed with lung adenocarcinoma (T1cN0M0, IA3) in May 2015 (Fig. S1A, B). After thoracoscopic resection, *EGFR* 19-Del was detected using an amplification refractory mutation system. No adjuvant therapy was performed.

In July 2016, the patient presented symptoms of progressive disease (PD) such as a progressive headache, while no abnormality was shown on magnetic resonance imaging (MRI) (Fig. S1C), so anxiolytic treatment was performed according to symptoms. Four months later, he presented clinical symptoms of intracranial hypertension, such as blurred vision, obvious dizziness, and syncope. Kamofsky performance status (KPS) score was 70. A lumbar puncture was performed to reduce intracranial pressure, and cerebrospinal fluid (CSF) was sent for pathological diagnosis confirming LM. From December 2016 to September 2017, he received erlotinib and AZD3759, and his symptoms were controlled. Meanwhile, next-generation sequencing (NGS) analysis of CSF confirmed *EGFR* 19-Del. Osimertinib treatment was then included, and the patient underwent a second CSF NGS, with only *EGFR* 19-Del detected. Until April 2019, the disease was stable with a continual decrease in carcinoembryonic antigen (CEA) levels and the relief of symptoms (KPS: 90). No progression was observed according to the image scan (Fig. S1D).

In May 2019, the patient suffered from PD and was treated with bevacizumab, nab-paclitaxel, and carboplatin. As carcinoembryonic antigen (CEA) levels increased sharply (Fig. 1A), he received osimertinib plus cabozantinib in December 2019; however, no symptomatic remission was observed. By a third CSF NGS, *EGFR*-*SEPT14* fusions were newly detected (Fig. 1B).

**Figure 1:**
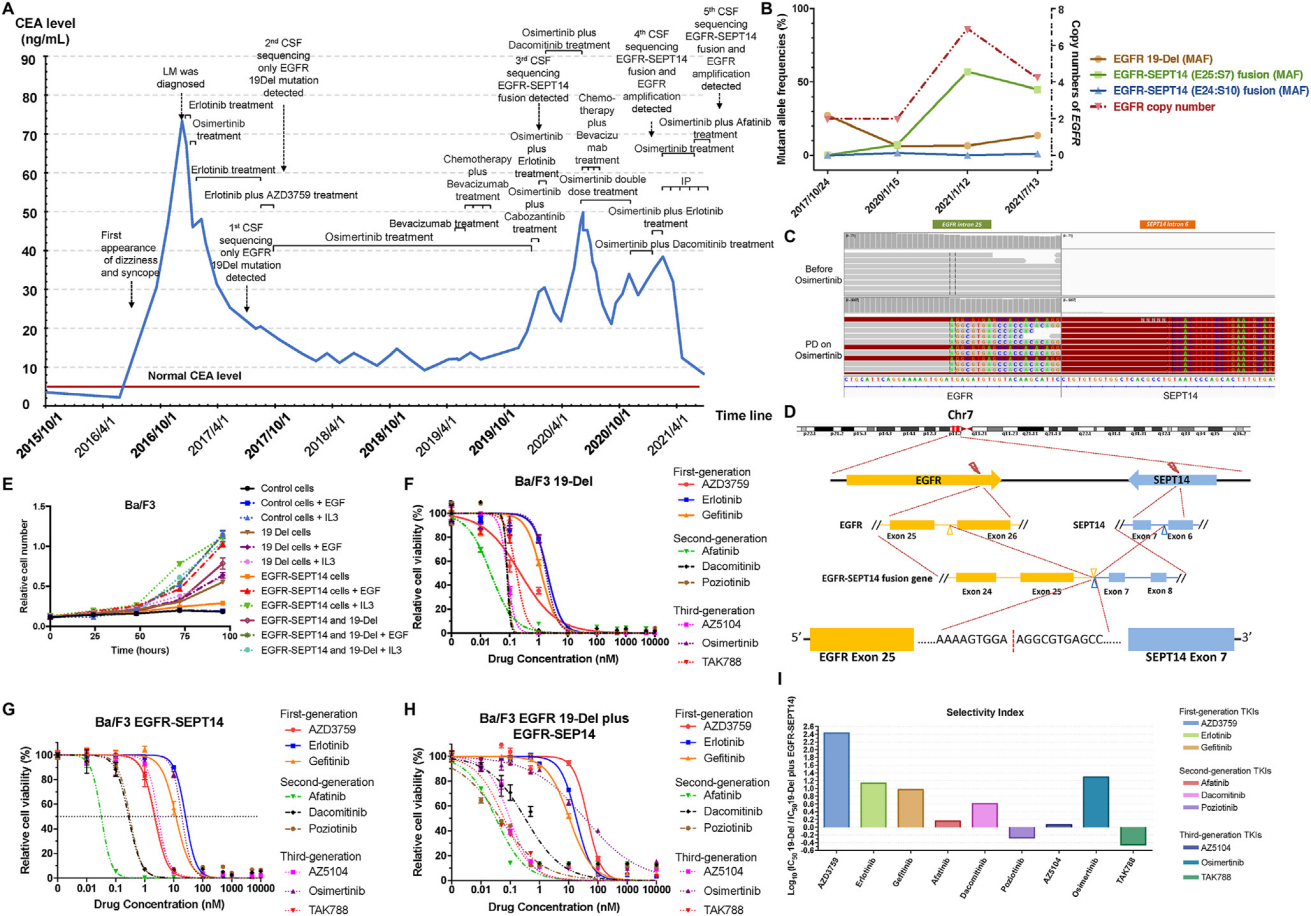
Identification of a novel acquired *EGFR-SEPT14* (E25:S7) fusion in a lung adenocarcinoma patient with LM and *EGFR-SEPT14* (E25:S7) fusion conferred drug resistance to certain EGFR TKIs. **(A)** The CEA level of the lung adenocarcinoma patient from the time of diagnosis until the last CSF sequencing. The normal CEA level and checkpoints of the treatment history were labeled. **(B)** The dynamic change of MAFs of the detected *EGFR* genetic alterations and copy numbers of *EGFR* amplification across the treatment history. **(C)** The detection of *EGFR-SEPT14* (E25:S7) fusion after PD on osimertinib. **(D)** The schematic of the structure of *EGFR-SEPT14* (E25:S7) fusion. Exons 1–25 of *EGFR* (yellow) were fused to exons 7–10 of *SEPT14* (blue) through intron 25 of *EGFR* and intron 6 of *SEPT14*. **(E)** The proliferation of Ba/F3 cells that stably expressed vector control, *EGFR* 19-Del, *EGFR-SEPT14* (E25:S7) fusion, or *EGFR* 19-Del plus *EGFR-SEPT14* (E25:S7) fusion in the presence/absence of EGF or IL3. **(F, G)** The growth–inhibitory curves of Ba/F3 cells expressing either *EGFR* 19-Del (F) or *EGFR-SEPT14* (E25:S7) fusion (G) in response to different EGFR TKIs. For *EGFR-SEPT14* (E25:S7) fusion, cells were stimulated with 50 ng/mL EGF. **(H)** The growth–inhibitory curves of Ba/F3 cells co-expressing *EGFR* 19-Del and *EGFR-SEPT14* (E25:S7) fusion in response to different EGFR TKIs. **(I)** The selectivity index (*i.e.*, the logarithmic transformation of IC_50_ ratio between solely expressing EGFR 19-Del and co-expressing *EGFR* 19-Del and *EGFR-SEPT14* fusion). for various EGFR TKIs.

We treated the patient with osimertinib plus erlotinib, but his disease conditions became worsened. From February to June 2020, he received dacomitinib plus osimertinib, resulting in noted improvement in his symptoms (KPS: 90) with a reduction in CEA level (Fig. 1A). However, in June 2020, the patient presented with headache and vomiting again, and the result of the increasing CEA suggested PD. Therefore, he then underwent 4 cycles of chemotherapy with bevacizumab, nab-paclitaxel, and carboplatin and maintained a double dose of osimertinib until PD in November 2020. By administering dacomitinib plus osimertinib, he experienced a short period of disease control. However, the patient suffered hearing dysesthesia and blurred vision (KPS: 40) soon, which displayed evidence of PD.

In January 2021, the fourth CSF NGS showed increased mutant allele frequency (MAF) of *EGFR*-*SEPT14* (from 7.2% to 57.0%), together with *EGFR* amplification (Fig. 1B). The patient was subjected to IP to treat LM and osimertinib for maintenance therapy. After 3 months, his CEA level decreased from 38.42 ng/mL to 12.36 ng/mL, accompanied by a relief of symptoms. No recurrence or abnormality was found according to the patient's last CT and MRI scan (Fig. S1E, F). Based on *in vitro* findings (results below), the patient received afatinib plus osimertinib for maintenance treatment in June 2021, and his CEA levels further dropped to 8.24 ng/mL (Fig. 1A). The fifth CSF NGS (July 2021) showed that the MAF of *EGFR-SEPT1*4 had decreased from 57.0% to 44.8% and *EGFR* copy number was decreased from 6.9 to 4.2 (Fig. 1B).

After initial TKI resistance, the patient acquired two forms of *EGFR-SEPT14* fusions. The first one fused *EGFR* exon-24 with *SEPT14* exon-10 (E24:S10), which was reported in glioblastomas^5^ and was unlikely to confer drug resistance due to its sensitivity to erlotinib^5^ and low MAF (Fig. 1B). The second one linked *EGFR* exon-25 to *SEPT14* exon-7 (E25:S7) (Fig. 1C, D), whose MAFs were well-corresponded to disease conditions (Fig. 1A, B), implying to be a novel TKI resistant mechanism. Simulated structural analyses revealed that EGFR-SEPT14 (E25:S7) and wild-type EGFR had similar secondary but distinct tertiary structures (Fig. S2A, B), suggesting that the fusion protein might have different functions and/or drug-binding capacities.

Next, we stably expressed *EGFR-SEPT14* (E25:S7) or EGFR 19-Del in Ba/F3 cells (Fig. S3). Compared with *EGFR* 19-Del that conferred IL3-independent growth, *EGFR-SEPT14* (E25:S7)-transfected cells could not proliferate in IL3-free media unless supplemented with EGF (Fig. 1E). We then investigated its drug sensitivity under EGF stimulation. Unlike *EGFR* 19-Del-transfected cells that were sensitive to all tested TKIs (Fig. 1F), *EGFR-SEPT14* (E25:S7)-transfected cells demonstrated significantly increased IC_50_ for multiple TKIs, especially first-generation TKIs and osimertinib (Fig. 1G and Table S2). Similar results were obtained by signaling analyses (Fig. S4).

To further simulate the pathological scenario, we co-transfected *EGFR* 19-Del and *EGFR-SEPT14* (E25:S7) into Ba/F3 cells, which could proliferate in the absence of IL3 and/or EGF (Fig. 1E). Notably, the co-transfected cells also maintained high sensitivity to some second- and third-generation TKIs but gained drug resistance to first-generation TKIs and osimertinib (Fig. 1H, I and Table S3).

Our results suggested that *EGFR-SEPT14* (E25:S7) was not oncogenic, but it can function together with *EGFR* 19-Del. Previous studies demonstrated that EGFR could form heterodimers to stimulate downstream signaling. Therefore, we suspected that EGFR-SEPT14 (E25:S7) could couple with EGFR 19-Del to form heterodimers, conferring oncogenicity and hindering the access of certain TKIs. Overall, we identified a novel *EGFR-SEPT14* (E25:S7) fusion as a resistant mechanism to EGFR TKIs. Despite resistance to first-generation TKIs and osimertinib, *EGFR-SEPT14* (E25:S7) responded well to second-generation and some third-generation TKIs.

## Ethics declaration

The patient included in this research signed an informed consent according to the Research Ethics Committee of The First Affiliated Hospital, School of Medicine, Zhejiang University.

## Author contributions

Wei-dong Wang: conceptualization, data curation, formal analysis, methodology, software, visualization, and roles/writing - original draft; Wang Lv: conceptualization, data curation, formal analysis, methodology, visualization, and roles/writing - original draft; Hui Wang: formal analysis, methodology, and visualization; Yang Xu: methodology, visualization, and roles/writing - original draft; Junrong Yan: formal analysis, methodology, and roles/writing - original draft; Han-Ming Shen: supervision, validation, and writing - review & editing; Li-qun Shan: conceptualization, funding acquisition, investigation, software, and writing - review & editing; Jian Hu: conceptualization, funding acquisition, project administration, resources, supervision, validation, and writing - review & editing.

## Conflict of interests

Yang Xu and Junrong Yan were employed by the company Nanjing Geneseeq Technology Inc. The remaining authors declare no potential conflict of interests.

## Funding

This study was supported by the Major Science & Technology Project of Zhejiang Province, China (No. 2020C03058), Key Disciplines of Traditional Chinese Medicine in Zhejiang Province, China (No. 2017-XKA33), Research Center of Pulmonary Oncology of Zhejiang Province, China (No. JBZX-202007), and Science and Technology Plan Project of Wenling, Zhejiang Province, China (No. 2020S0180105s).

## Data availability

The human sequence data generated in this study are not publicly available due to patient privacy requirements but are available upon reasonable request from the corresponding author. Other data generated in this study are available within the article and its supplementary data files.

## Figures and Tables

**figs1:**
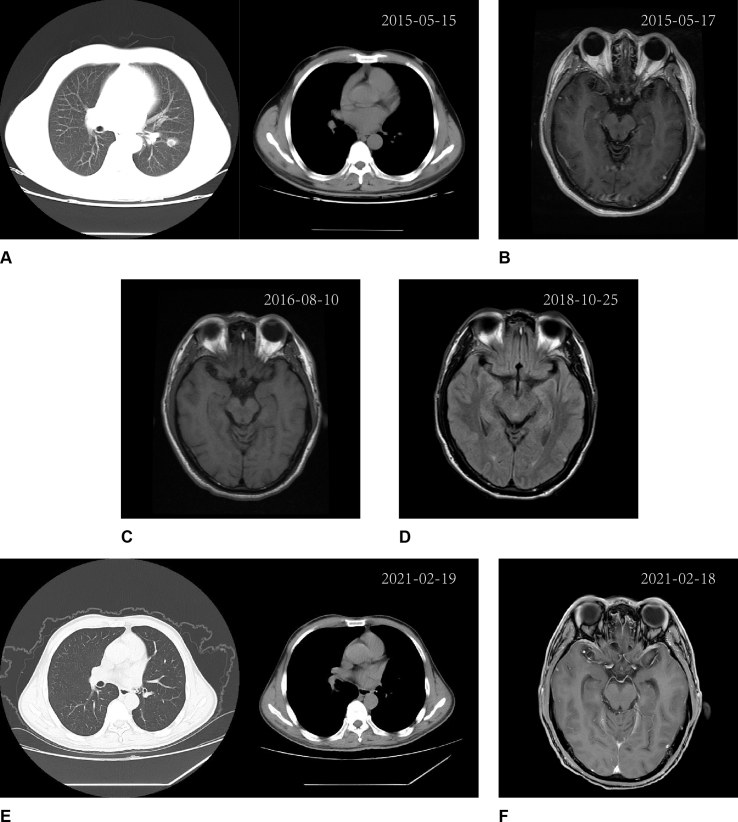


**figs2:**
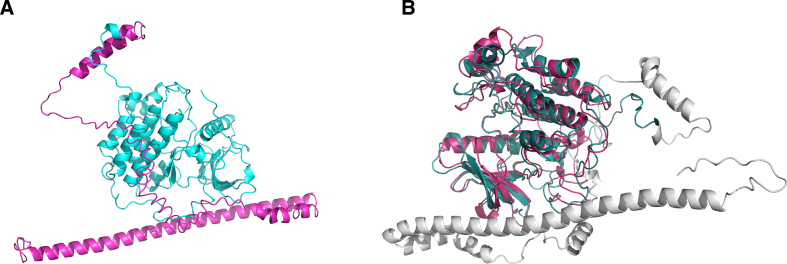


**figs3:**
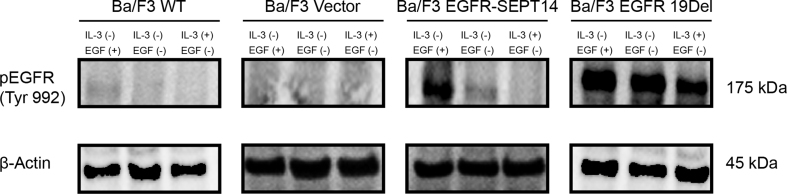


**figs4:**