# Supplementary material for: A novel acquired EGFR-SEPT14 fusion confers differential drug resistance to EGFR inhibitors in lung adenocarcinoma
Source: Genes Dis. 2023 Apr 24;10(6):2241–4. doi: 10.1016/j.gendis.2023.02.019 (PMC10404941; doi:10.1016/j.gendis.2023.02.019)
Supplement: Multimedia component 1 [file mmc1.docx]

**Supplementary methods**

***Study design and sample collection***

An NSCLC patient was diagnosed at The First Affiliated Hospital on May 15, 2015, and the patient received various EGFR TKI-base treatments. The patient has signed a written consent statement for publication of related clinical results and analyses. The serial cerebrospinal fluid (CSF) specimens were collected during the course of treatment and were subjected to next-generation sequencing (NGS) analysis of 139 lung cancer-relevant genes [1, 2] to identify any potential drug-resistant mechanisms.

***DNA Extraction, library preparation, and NGS***

Cell-free DNA from CSF was extracted using QIAamp circulating nucleic acid kit (Qiagen, USA) based on the manufacturer’s instructions, and the quantiﬁcation of DNA was performed by Qubit 3.0 dsDNA HS assay (Life Technologies, Carlsbad, CA, USA). The detailed library preparation and NGS procedure was conducted following previously reported approaches [1, 2]. Briefly, DNA was fragmented and subjected to the process of end-repairing, A-tailing, indexed-adapter ligation, size selection, and PCR ampliﬁcation, and the DNA library was then hybridized with customized xGen lockdown probes (Integrated DNA Technologies, Coralville, IA, USA) for predeﬁned 139 cancer-associated genes. Then, enriched libraries were ampliﬁed and subjected for next-generation sequencing on the platform of Illumina Hiseq4000 (Illumina, San Diego, CA, USA).

Trimmomatic was used for raw FASTQ ﬁle quality control [3], and reads were mapped to the reference human genome (hg19) using BWAmem (https://github.com/lh3/bwa/tree/master/ bwakit) [4]. Local realignment around the indels and base quality score recalibration was performed using the Genome Analysis Toolkit GATK 3.4.0 (https://software.broadinstitute. org/gatk/) [5]. The somatic mutation was detected with VarScan2. Genomic fusions were found by FACTERA [6], and copy number variations (CNVs) were identiﬁed using ADTEx (http:// adtex.sourceforge.net) using default parameters.

***Computational structure modeling***

The fusion protein was modeled using I-TASSER, including the connections between EGFR intracellular domains and SEPT14. The crystal structure of the EGFR fusion protein was visualized by PyMOL (http://www.pymol.org/pymol).

***Cell culture and retroviral infection***

Ba/F3 cells were maintained in RPMI-1640 media (CORNING, Mediatech, Inc., Manassas, VA, USA) supplemented with 10 ng/ml mouse interleukin-3 (IL-3) (Cell Signaling Technology, Danvers, MA) and 10% fetal bovine serum (PAN-Biotech, Aidenbach, Germany) in a 5% CO_2_ atmosphere at 37 °C. EGFR TKIs were purchased from Selleck Chemicals (Houston, TX).

Commercial retroviruses expressing the *EGFR*-*SEPT14* (E25:S7) fusion gene or *EGFR* 19-Del were supplied by Genewiz Co. Ltd. (Suzhou, China). The full-length *EGFR*-*SEPT14* (E25:S7) fusion and *EGFR* 19-Del cDNA were subcloned into the pGWLV11 expression vector. Ba/F3 cells were retrovirally infected and selected following standard protocols [7]. The co-transfection was conducted as followed: firstly, we selected Ba/F3 cells which stably expressed EGFR-SEPT14 (E25:S7) by western blot. Then, those cells were further retrovirally infected with EGFR 19-Del cDNA and selected and confirmed by western blot.

***Cell proliferation and growth inhibition assay***

Ba/F3 cells were transfected with vector control, *EGFR*-*SEPT14* (E25:S7), or *EGFR* 19-Del and then were plated at 5,000 cells/well in a 96-well plate in the presence or absence of IL-3 or epidermal growth factor (EGF). Cells were collected at 36 h and 72 h after seeding and assessed by Cell Counting Kit-8 (CCK-8) assays (Dojindo, Japan) as previously described [8].

Ba/F3 cells stably expressing only *EGFR*-*SEPT14* (E25:S7), only *EGFR* 19-Del, or both of the constructs were seeded in 96-well plates at a density of 2,500 cells/well. EGF was added for cells only expressing *EGFR-SEPT14* (E25:S7). Dimethyl sulfoxide (DMSO) or TKIs were added at the indicated concentration to the cells and cultured for 72 h. The CCK-8 assay was used to determine cell growth inhibition by following the previously established protocol [9].

***Western blot***

*EGFR*-*SEPT14* fusion or *EGFR* 19-Del transfected Ba/F3 cells were plated in 6-well plates with 50 ng/mL EGF and treated with the indicated concentrations of TKIs or DMSO for 4 h. Cells were then collected and lysed by RIPA buffer (Beyotime, Shanghai, China) supplemented with protease and phosphatase inhibitors. The western blot assay was performed as previously reported [10] using the antibodies listed in **Table** S**1**.

***Specific diagnosis and treatment process***

The patient was a 51-year-old male nonsmoker whose lower lobe of the left lung exhibited a mixed glass ground opacity with a diameter of 2 cm during a routine physical examination on May 15, 2015, no metastasis was found before operation according to radiology (Supplementary Figure 1 A and B). Then, he underwent video-assisted thoracoscopic left lower lobe resection with systemic nodal dissection on May 21, 2015. Postoperative pathology revealed a 2.2-cm middle-differentiated adenocarcinoma with the major component of acinous and papillary growth. No pleural invasion or lymph node metastasis was reported (T1cN0M0, IA3). The EGFR mutation in the tumor sample was evaluated by an amplification refractory mutation system and indicated an EGFR 19-Del mutation. No adjuvant therapy was performed.

In July 2016, the patient presented with a progressive headache, while no abnormality was shown on magnetic resonance imaging (MRI) (Supplementary Figure 1C), so anxiolytic treatment was performed according to symptoms. Four months later, he presented clinical symptoms of intracranial hypertension, such as blurred vision, obvious dizziness, and syncope. Kamofsky performance status (KPS) score was 70 and Zubrod-ECOG-WHO performance status (ZPS) score was 2. Lumbar puncture was performed to reduce intracranial pressure, and CSF was sent for pathological diagnosis confirming LM. From December 2016 to September 2017, the patient underwent erlotinib monotherapy (150 mg once daily administered orally) and erlotinib plus AZD3759 (200mg twice a day), and the symptoms were controlled (KPS: 80, ZPS: 1). Meanwhile, his CSF was subjected to NGS, in which only the EGFR 19-Del was detected. To further control the disease, the patient’s treatment plan was altered to include osimertinib (80 mg once daily administered orally) on September 22, 2017. In October 2017, the patient underwent 2nd CSF NGS, which showed EGFR 19-Del (27.3%). From then on to April 2019, the disease was stable with a continual decrease in carcinoembryonic antigen (CEA) levels and the relief of symptoms (KPS: 90, ZPS: 1). No progression was observed according to the image scan (Supplementary Figure 1D). In May 2019, the patient suffered a worsening headache suggesting progressive disease (PD) and was treated with bevacizumab (15 mg/kg, intravenously, on day 1 of each 3-week cycle) or bevacizumab plus nab-paclitaxel (400mg, intravenously, on day 1 of each 3-week cycle) and carboplatin (at an area of the concentration-time curve of 6 mg/ml/min, intravenously, on day 1 of each 3-week cycle). Because the disease was resistant to chemotherapy and the CEA level increased sharply, the patient was started on osimertinib (80 mg per day) plus cabozantinib (60 mg once daily administered orally) on December 28, 2019; however, no symptomatic remission was observed (KPS: 70, ZPS: 2). Thus, he underwent the third CSF NGS, and aside from EGFR 19-Del (6.4%), a novel EGFR-SEPT14 fusion was detected. The patient was found to harbor two uncommon subtypes of EGFR-SEPT14 fusion, including EGFR exon 25 fused to SEPT14 exon 7 (7.2%) with breakpoints in EGFR intron 25 and SEPT14 intron 6, as well as exon 24 on EGFR fused to exon 10 on SEPT14 (1.7%).

Considering that the patient harbored the EGFR-SEPT14 fusion, he started osimertinib (80 mg per day) combined with erlotinib (150 mg per day) on January 15, 2020, but the disease worsened (KPS: 60, ZPS: 2). From February to May 2020, the patient was treated with dacomitinib plus osimertinib and noted improvement in his symptoms (KPS: 90, ZPS: 1) with a reduction in CEA level. However, in June 2020, the patient presented with headache and vomiting again, and the result of the increasing CEA suggested PD. Therefore, he underwent 4 cycles of chemotherapy with bevacizumab plus nab-paclitaxel and carboplatin and was maintained at a double-dose of osimertinib (160 mg per day) until November 2020 when the disease progressed again. The patient was administered dacomitinib (45 mg once daily administered orally) plus osimertinib (80 mg per day) for the second time. After a short period of disease control, the patient suffered hearing dysesthesia and blurred vision (KPS: 40, ZPS: 3), which displayed evidence of PD. On January 12, 2020, he underwent a fourth CSF NGS, and the results indicated that the mutant frequency of EGFR-SEPT14 fusion increased significantly from 7.2% to 57.0% and had EGFR amplification with a copy number of 6.9. Because of the deterioration of disease and constantly increased level of CEA, the patient was subjected to intrathecal pemetrexed (IP) (50 mg, on day 1 of each 3-week cycle) to treat his LM and osimertinib (80 mg per day) for maintenance therapy. After 3 months of complex therapy, his CEA level decreased from 38.42 ng/mL to 12.36 ng/mL accompanied by a relief of symptoms (KPS: 90, ZPS: 1). No recurrence or abnormality was found according to the patient’s last CT and MRI scan (Supplementary Figure 1E-F). Based on our in vitro findings, afatinib (40 mg once daily administered orally) was advised in combination with osimertinib for maintenance treatment in June 2021, and the patient continued to receive IP. His CEA further dropped to 8.24 ng/mL, which was the lowest level since his disease metastasized. On July 13, 2021, his fifth CSF NGS results showed that the EGFR-SEPT14 fusion frequency had decreased from 57.0% to 44.8%, and the EGFR copy number was decreased to 4.2 ng/m.

***The insert sequence for vector construction regarding EGFR 19-Del***

ATGCGACCCTCCGGGACGGCCGGGGCAGCGCTCCTGGCGCTGCTGGCTGCGCTCTGCCCGGCGAGTCGGGCTCTGGAGGAAAAGAAAGTTTGCCAAGGCACGAGTAACAAGCTCACGCAGTTGGGCACTTTTGAAGATCATTTTCTCAGCCTCCAGAGGATGTTCAATAACTGTGAGGTGGTCCTTGGGAATTTGGAAATTACCTATGTGCAGAGGAATTATGATCTTTCCTTCTTAAAGACCATCCAGGAGGTGGCTGGTTATGTCCTCATTGCCCTCAACACAGTGGAGCGAATTCCTTTGGAAAACCTGCAGATCATCAGAGGAAATATGTACTACGAAAATTCCTATGCCTTAGCAGTCTTATCTAACTATGATGCAAATAAAACCGGACTGAAGGAGCTGCCCATGAGAAATTTACAGGAAATCCTGCATGGCGCCGTGCGGTTCAGCAACAACCCTGCCCTGTGCAACGTGGAGAGCATCCAGTGGCGGGACATAGTCAGCAGTGACTTTCTCAGCAACATGTCGATGGACTTCCAGAACCACCTGGGCAGCTGCCAAAAGTGTGATCCAAGCTGTCCCAATGGGAGCTGCTGGGGTGCAGGAGAGGAGAACTGCCAGAAACTGACCAAAATCATCTGTGCCCAGCAGTGCTCCGGGCGCTGCCGTGGCAAGTCCCCCAGTGACTGCTGCCACAACCAGTGTGCTGCAGGCTGCACAGGCCCCCGGGAGAGCGACTGCCTGGTCTGCCGCAAATTCCGAGACGAAGCCACGTGCAAGGACACCTGCCCCCCACTCATGCTCTACAACCCCACCACGTACCAGATGGATGTGAACCCCGAGGGCAAATACAGCTTTGGTGCCACCTGCGTGAAGAAGTGTCCCCGTAATTATGTGGTGACAGATCACGGCTCGTGCGTCCGAGCCTGTGGGGCCGACAGCTATGAGATGGAGGAAGACGGCGTCCGCAAGTGTAAGAAGTGCGAAGGGCCTTGCCGCAAAGTGTGTAACGGAATAGGTATTGGTGAATTTAAAGACTCACTCTCCATAAATGCTACGAATATTAAACACTTCAAAAACTGCACCTCCATCAGTGGCGATCTCCACATCCTGCCGGTGGCATTTAGGGGTGACTCCTTCACACATACTCCTCCTCTGGATCCACAGGAACTGGATATTCTGAAAACCGTAAAGGAAATCACAGGGTTTTTGCTGATTCAGGCTTGGCCTGAAAACAGGACGGACCTCCATGCCTTTGAGAACCTAGAAATCATACGCGGCAGGACCAAGCAACATGGTCAGTTTTCTCTTGCAGTCGTCAGCCTGAACATAACATCCTTGGGATTACGCTCCCTCAAGGAGATAAGTGATGGAGATGTGATAATTTCAGGAAACAAAAATTTGTGCTATGCAAATACAATAAACTGGAAAAAACTGTTTGGGACCTCCGGTCAGAAAACCAAAATTATAAGCAACAGAGGTGAAAACAGCTGCAAGGCCACAGGCCAGGTCTGCCATGCCTTGTGCTCCCCCGAGGGCTGCTGGGGCCCGGAGCCCAGGGACTGCGTCTCTTGCCGGAATGTCAGCCGAGGCAGGGAATGCGTGGACAAGTGCAACCTTCTGGAGGGTGAGCCAAGGGAGTTTGTGGAGAACTCTGAGTGCATACAGTGCCACCCAGAGTGCCTGCCTCAGGCCATGAACATCACCTGCACAGGACGGGGACCAGACAACTGTATCCAGTGTGCCCACTACATTGACGGCCCCCACTGCGTCAAGACCTGCCCGGCAGGAGTCATGGGAGAAAACAACACCCTGGTCTGGAAGTACGCAGACGCCGGCCATGTGTGCCACCTGTGCCATCCAAACTGCACCTACGGATGCACTGGGCCAGGTCTTGAAGGCTGTCCAACGAATGGGCCTAAGATCCCGTCCATCGCCACTGGGATGGTGGGGGCCCTCCTCTTGCTGCTGGTGGTGGCCCTGGGGATCGGCCTCTTCATGCGAAGGCGCCACATCGTTCGGAAGCGCACGCTGCGGAGGCTGCTGCAGGAGAGGGAGCTTGTGGAGCCTCTTACACCCAGTGGAGAAGCTCCCAACCAAGCTCTCTTGAGGATCTTGAAGGAAACTGAATTCAAAAAGATCAAAGTGCTGGGCTCCGGTGCGTTCGGCACGGTGTATAAGGGACTCTGGATCCCAGAAGGTGAGAAAGTTAAAATTCCCGTCGCTATCAAAACATCTCCGAAAGCCAACAAGGAAATCCTCGATGAAGCCTACGTGATGGCCAGCGTGGACAACCCCCACGTGTGCCGCCTGCTGGGCATCTGCCTCACCTCCACCGTGCAGCTCATCACGCAGCTCATGCCCTTCGGCTGCCTCCTGGACTATGTCCGGGAACACAAAGACAATATTGGCTCCCAGTACCTGCTCAACTGGTGTGTGCAGATCGCAAAGGGCATGAACTACTTGGAGGACCGTCGCTTGGTGCACCGCGACCTGGCAGCCAGGAACGTACTGGTGAAAACACCGCAGCATGTCAAGATCACAGATTTTGGGCTGGCCAAACTGCTGGGTGCGGAAGAGAAAGAATACCATGCAGAAGGAGGCAAAGTGCCTATCAAGTGGATGGCATTGGAATCAATTTTACACAGAATCTATACCCACCAGAGTGATGTCTGGAGCTACGGGGTGACTGTTTGGGAGTTGATGACCTTTGGATCCAAGCCATATGACGGAATCCCTGCCAGCGAGATCTCCTCCATCCTGGAGAAAGGAGAACGCCTCCCTCAGCCACCCATATGTACCATCGATGTCTACATGATCATGGTCAAGTGCTGGATGATAGACGCAGATAGTCGCCCAAAGTTCCGTGAGTTGATCATCGAATTCTCCAAAATGGCCCGAGACCCCCAGCGCTACCTTGTCATTCAGGGGGATGAAAGAATGCATTTGCCAAGTCCTACAGACTCCAACTTCTACCGTGCCCTGATGGATGAAGAAGACATGGACGACGTGGTGGATGCCGACGAGTACCTCATCCCACAGCAGGGCTTCTTCAGCAGCCCCTCCACGTCACGGACTCCCCTCCTGAGCTCTCTGAGTGCAACCAGCAACAATTCCACCGTGGCTTGCATTGATAGAAATGGGCTGCAAAGCTGTCCCATCAAGGAAGACAGCTTCTTGCAGCGATACAGCTCAGACCCCACAGGCGCCTTGACTGAGGACAGCATAGACGACACCTTCCTCCCAGTGCCTGAATACATAAACCAGTCCGTTCCCAAAAGGCCCGCTGGCTCTGTGCAGAATCCTGTCTATCACAATCAGCCTCTGAACCCCGCGCCCAGCAGAGACCCACACTACCAGGACCCCCACAGCACTGCAGTGGGCAACCCCGAGTATCTCAACACTGTCCAGCCCACCTGTGTCAACAGCACATTCGACAGCCCTGCCCACTGGGCCCAGAAAGGCAGCCACCAAATTAGCCTGGACAACCCTGACTACCAGCAGGACTTCTTTCCCAAGGAAGCCAAGCCAAATGGCATCTTTAAGGGCTCCACAGCTGAAAATGCAGAATACCTAAGGGTCGCGCCACAAAGCAGTGAATTTATTGGAGCATGA

***The insert sequence for vector construction regarding EGRF-SEPT14(E25:S7)***

ATGCGACCCTCCGGGACGGCCGGGGCAGCGCTCCTGGCGCTGCTGGCTGCGCTCTGCCCGGCGAGTCGGGCTCTGGAGGAAAAGAAAGTTTGCCAAGGCACGAGTAACAAGCTCACGCAGTTGGGCACTTTTGAAGATCATTTTCTCAGCCTCCAGAGGATGTTCAATAACTGTGAGGTGGTCCTTGGGAATTTGGAAATTACCTATGTGCAGAGGAATTATGATCTTTCCTTCTTAAAGACCATCCAGGAGGTGGCTGGTTATGTCCTCATTGCCCTCAACACAGTGGAGCGAATTCCTTTGGAAAACCTGCAGATCATCAGAGGAAATATGTACTACGAAAATTCCTATGCCTTAGCAGTCTTATCTAACTATGATGCAAATAAAACCGGACTGAAGGAGCTGCCCATGAGAAATTTACAGGAAATCCTGCATGGCGCCGTGCGGTTCAGCAACAACCCTGCCCTGTGCAACGTGGAGAGCATCCAGTGGCGGGACATAGTCAGCAGTGACTTTCTCAGCAACATGTCGATGGACTTCCAGAACCACCTGGGCAGCTGCCAAAAGTGTGATCCAAGCTGTCCCAATGGGAGCTGCTGGGGTGCAGGAGAGGAGAACTGCCAGAAACTGACCAAAATCATCTGTGCCCAGCAGTGCTCCGGGCGCTGCCGTGGCAAGTCCCCCAGTGACTGCTGCCACAACCAGTGTGCTGCAGGCTGCACAGGCCCCCGGGAGAGCGACTGCCTGGTCTGCCGCAAATTCCGAGACGAAGCCACGTGCAAGGACACCTGCCCCCCACTCATGCTCTACAACCCCACCACGTACCAGATGGATGTGAACCCCGAGGGCAAATACAGCTTTGGTGCCACCTGCGTGAAGAAGTGTCCCCGTAATTATGTGGTGACAGATCACGGCTCGTGCGTCCGAGCCTGTGGGGCCGACAGCTATGAGATGGAGGAAGACGGCGTCCGCAAGTGTAAGAAGTGCGAAGGGCCTTGCCGCAAAGTGTGTAACGGAATAGGTATTGGTGAATTTAAAGACTCACTCTCCATAAATGCTACGAATATTAAACACTTCAAAAACTGCACCTCCATCAGTGGCGATCTCCACATCCTGCCGGTGGCATTTAGGGGTGACTCCTTCACACATACTCCTCCTCTGGATCCACAGGAACTGGATATTCTGAAAACCGTAAAGGAAATCACAGGGTTTTTGCTGATTCAGGCTTGGCCTGAAAACAGGACGGACCTCCATGCCTTTGAGAACCTAGAAATCATACGCGGCAGGACCAAGCAACATGGTCAGTTTTCTCTTGCAGTCGTCAGCCTGAACATAACATCCTTGGGATTACGCTCCCTCAAGGAGATAAGTGATGGAGATGTGATAATTTCAGGAAACAAAAATTTGTGCTATGCAAATACAATAAACTGGAAAAAACTGTTTGGGACCTCCGGTCAGAAAACCAAAATTATAAGCAACAGAGGTGAAAACAGCTGCAAGGCCACAGGCCAGGTCTGCCATGCCTTGTGCTCCCCCGAGGGCTGCTGGGGCCCGGAGCCCAGGGACTGCGTCTCTTGCCGGAATGTCAGCCGAGGCAGGGAATGCGTGGACAAGTGCAACCTTCTGGAGGGTGAGCCAAGGGAGTTTGTGGAGAACTCTGAGTGCATACAGTGCCACCCAGAGTGCCTGCCTCAGGCCATGAACATCACCTGCACAGGACGGGGACCAGACAACTGTATCCAGTGTGCCCACTACATTGACGGCCCCCACTGCGTCAAGACCTGCCCGGCAGGAGTCATGGGAGAAAACAACACCCTGGTCTGGAAGTACGCAGACGCCGGCCATGTGTGCCACCTGTGCCATCCAAACTGCACCTACGGATGCACTGGGCCAGGTCTTGAAGGCTGTCCAACGAATGGGCCTAAGATCCCGTCCATCGCCACTGGGATGGTGGGGGCCCTCCTCTTGCTGCTGGTGGTGGCCCTGGGGATCGGCCTCTTCATGCGAAGGCGCCACATCGTTCGGAAGCGCACGCTGCGGAGGCTGCTGCAGGAGAGGGAGCTTGTGGAGCCTCTTACACCCAGTGGAGAAGCTCCCAACCAAGCTCTCTTGAGGATCTTGAAGGAAACTGAATTCAAAAAGATCAAAGTGCTGGGCTCCGGTGCGTTCGGCACGGTGTATAAGGGACTCTGGATCCCAGAAGGTGAGAAAGTTAAAATTCCCGTCGCTATCAAGGAATTAAGAGAAGCAACATCTCCGAAAGCCAACAAGGAAATCCTCGATGAAGCCTACGTGATGGCCAGCGTGGACAACCCCCACGTGTGCCGCCTGCTGGGCATCTGCCTCACCTCCACCGTGCAGCTCATCACGCAGCTCATGCCCTTCGGCTGCCTCCTGGACTATGTCCGGGAACACAAAGACAATATTGGCTCCCAGTACCTGCTCAACTGGTGTGTGCAGATCGCAAAGGGCATGAACTACTTGGAGGACCGTCGCTTGGTGCACCGCGACCTGGCAGCCAGGAACGTACTGGTGAAAACACCGCAGCATGTCAAGATCACAGATTTTGGGCTGGCCAAACTGCTGGGTGCGGAAGAGAAAGAATACCATGCAGAAGGAGGCAAAGTGCCTATCAAGTGGATGGCATTGGAATCAATTTTACACAGAATCTATACCCACCAGAGTGATGTCTGGAGCTACGGGGTGACTGTTTGGGAGTTGATGACCTTTGGATCCAAGCCATATGACGGAATCCCTGCCAGCGAGATCTCCTCCATCCTGGAGAAAGGAGAACGCCTCCCTCAGCCACCCATATGTACCATCGATGTCTACATGATCATGGTCAAGTGCTGGATGATAGACGCAGATAGTCGCCCAAAGTTCCGTGAGTTGATCATCGAATTCTCCAAAATGGCCCGAGACCCCCAGCGCTACCTTGTCATTCAGGGGGATGAAAGAATGCATTTGCCAAGTCCTACAGACTCCAACTTCTACCGTGCCCTGATGGATGAAGAAGACATGGACGACGTGGTGGATGCCGACGAGTACCTCATCCCACAGCAGGGCTTCTTCAGCAGCCCCTCCACGTCACGGACTCCCCTCCTGAGCTCTCTGGGGCTGTTACCCTTTGCTGTGGTAGGGAGTACAGATGAAGTGAAAGTTGGAAAAAGGATGGTCAGAGGCCGTCACTACCCTTGGGGAGTTTTGCAAGTGGAAAATGAAAATCACTGTGACTTCGTTAAGCTCCGAGATATGCTTCTTTGTACCAATATGGAAAATCTAAAAGAAAAAACCCACACTCAGCACTATGAATGTTATAGGTACCAAAAACTGCAGAAAATGGGCTTTACAGATGTGGGTCCAAACAACCAGCCAGTTAGTTTTCAAGAAATCTTTGAAGCCAAAAGACAAGAGTTCTATGATCAATGTCAGAGGGAAGAAGAAGAGTTGAAACAGAGATTTATGCAGCGAGTCAAGGAGAAAGAAGCAACATTTAAAGAAGCTGAAAAAGAGCTGCAGGACAAGTTCGAGCATCTTAAAATGATTCAACAGGAGGAGATAAGGAAGCTCGAGGAAGAGAAAAAACAACTGGAAGGAGAAATCATAGATTTTTATAAAATGAAAGCTGCCTCCGAAGCACTGCAGACTCAGCTGAGCACCGATACAAAGAAAGACAAACATCGTAAGAAATAA

**Supplementary tables and figures**

**Table S1** Detailed information of the utilized Western blot antibodies.

| **Antibody** | **Source** | **Manufacturer (Cat. #)** | **Working concentration** |
| --- | --- | --- | --- |
| Phospho-AKT (Thr308) | Rabbit | Cell Signaling Technology (#4056) | 1:1000 |
| AKT | Rabbit | Cell Signaling Technology (#9272) | 1:1000 |
| β-Actin | Mouse | Cell Signaling Technology (#3700) | 1:1000 |
| Phospho-EGFR (Tyr992) | Rabbit | Cell Signaling Technology (#2235) | 1:1000 |
| EGFR | Rabbit | Cell Signaling Technology (#4267) | 1:1000 |
| Phospho-ERK1/2 (Thr202/Tyr204) | Rabbit | Cell Signaling Technology (#9101) | 1:1000 |
| ERK1/2 | Rabbit | Cell Signaling Technology (#4695) | 1:1000 |
| Phospho-STAT5 (Tyr694) | Rabbit | Cell Signaling Technology (#9314) | 1:1000 |
| STAT5 | Rabbit | Cell Signaling Technology (#94205) | 1:1000 |

**Table S2** IC50 of various EGFR TKIs in *EGFR* 19-Del transfected Ba/F3 cells or in EGF stimulated *EGFR-SEPT14* transfected Ba/F3 cells.

| **EGFR-TKI** | **IC50 (nM)** | |
| --- | --- | --- |
|  | **19-Del** | **EGFR-SEPT14  (with EGF stimulation)** |
| **First-generation** |  |  |
| AZD3759 | 0.22 | 2.19 |
| Erlotinib | 2.02 | 25.4 |
| Gefitinib | 1.23 | 11.84 |
| **Second-generation** |  |  |
| Afatinib | 0.02 | 0.03 |
| Dacomitinib | 0.08 | 0.28 |
| Poziotinib | 0.08 | 0.31 |
| **Third-generation** |  |  |
| AZ5104 | 0.09 | 2.94 |
| Osimertinib | 1.79 | 18.99 |
| TAK788 | 0.17 | 2.36 |

**Table S3** IC50 of various EGFR TKIs in *EGFR* 19-Del and *EGFR-SEPT14* co-transfected Ba/F3 cells.

| **EGFR-TKI** | **IC50 (nM)** |
| --- | --- |
|  | **19-Del plus EGFR-SEPT14** |
| **First-generation** |  |
| AZD3759 | 62.33 |
| Erlotinib | 27.75 |
| Gefitinib | 11.39 |
| **Second-generation** |  |
| Afatinib | 0.032 |
| Dacomitinib | 0.324 |
| Poziotinib | 0.045 |
| **Third-generation** |  |
| AZ5104 | 0.097 |
| Osimertinib | 35.18 |
| TAK788 | 0.061 |


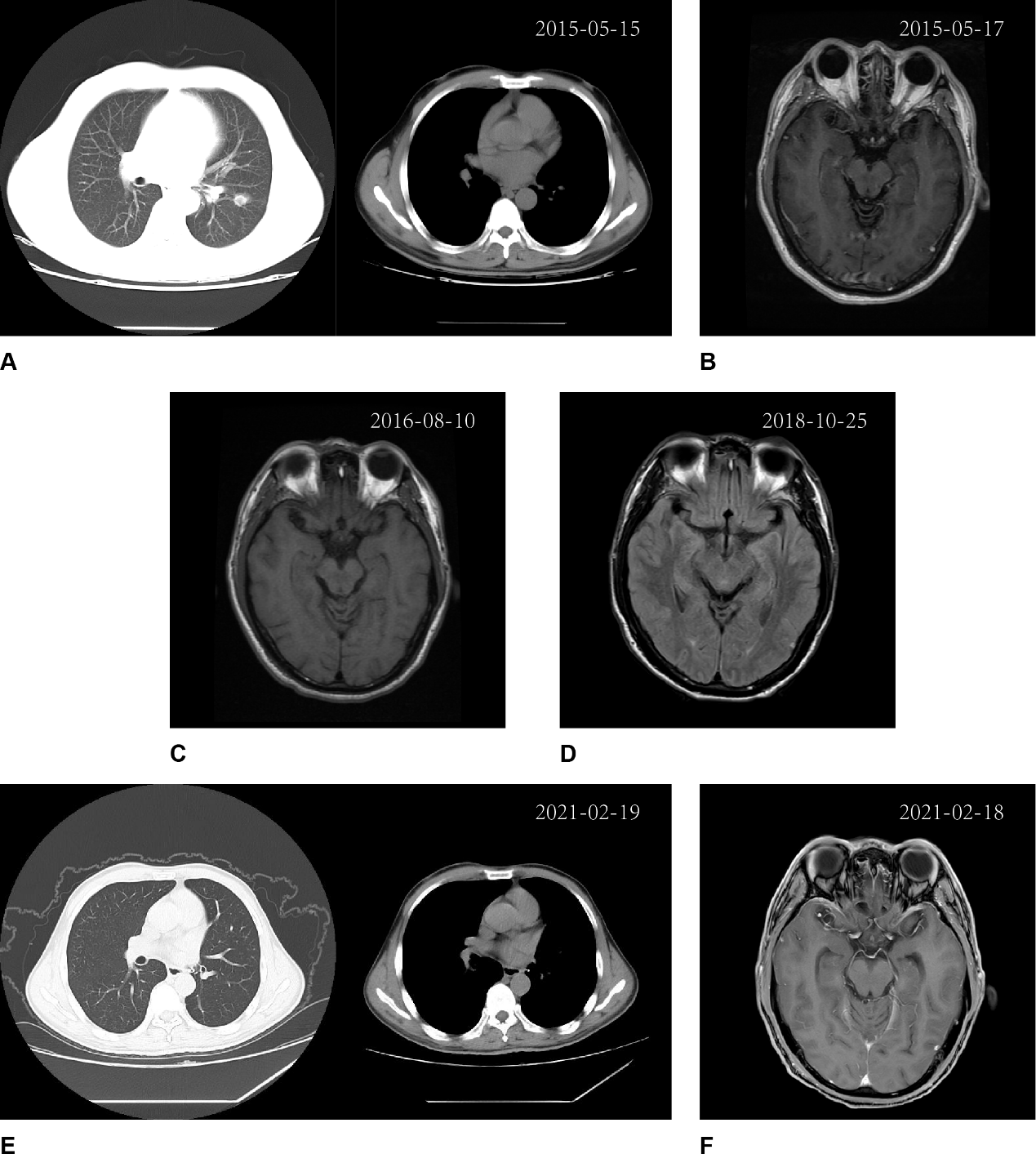


**Fig. S1 Clinical radiological imaging of this patient.**

**Fig. S2 The homology model of EGFR-SEPT14 (E25:S7)** **fusion protein.** **A** The homology model of EGFR-SEPT14 (E25:S7) fusion protein. The EGFR part was shown in cyan while the SEPT14 part was shown in magenta. **B** The comparison of molecular dynamics-simulated structures between WT EGFR and EGFR-SEPT14 (E25:S7) fusion proteins. The WT EGFR was shown in magenta; the EGFR part of the fusion protein was shown in dark green while and the SEPT14 part of the fusion protein was shown in gray.


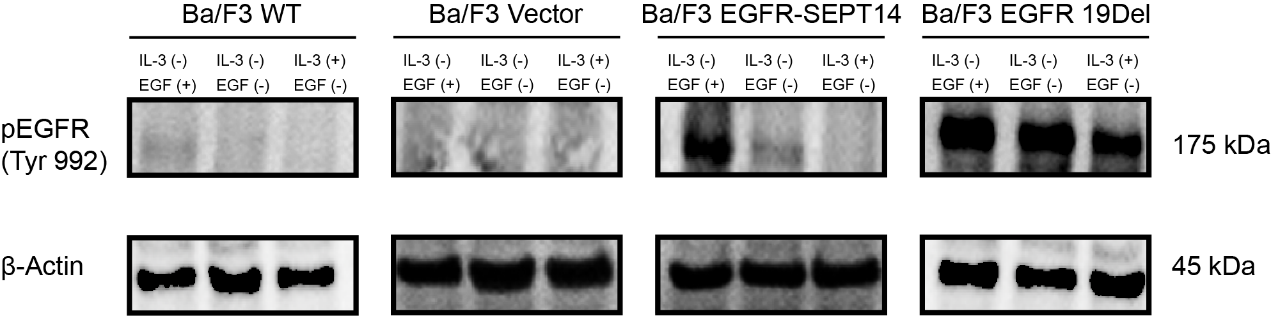


**Fig. S3** **The phosphorylation level of EGFR (Tyr 992) in wild-type Ba/F3 cells and Ba/F3 cells transfected with vector, *EGFR-SEPT14* (E25:S7)** **fusion and 19-Del in RPMI-1640 media with or without EGF and IL-3.**

**Fig. S4** **The drug sensitivity of *EGFR-SEPT14* (E25:S7)** **fusion to various EGFR TKIs.** (**A**-**C**) Inhibition of the phosphorylation of EGFR and downstream proteins by EGFR-TKIs in Ba/F3 cells harboring *EGFR-SEPT14* fusion by first-generation (**A**), second-generation (**B**), and third-generation TKIs (**C**) in the presence of EGF stimulation.

**References:**

1. Zheng MM, Li YS, Tu HY, Jiang BY, Yang JJ, Zhou Q, Xu CR, Yang XR, Wu YL: **Genotyping of Cerebrospinal Fluid Associated With Osimertinib Response and Resistance for Leptomeningeal Metastases in EGFR-Mutated NSCLC**. *J Thorac Oncol* 2021, **16**(2):250-258.

2. Zhang C, Zhang J, Xu FP, Wang YG, Xie Z, Su J, Dong S, Nie Q, Shao Y, Zhou Q *et al*: **Genomic Landscape and Immune Microenvironment Features of Preinvasive and Early Invasive Lung Adenocarcinoma**. *J Thorac Oncol* 2019, **14**(11):1912-1923.

3. Bolger AM, Lohse M, Usadel B: **Trimmomatic: a flexible trimmer for Illumina sequence data**. *Bioinformatics* 2014, **30**(15):2114-2120.

4. Li H, Durbin R: **Fast and accurate short read alignment with Burrows-Wheeler transform**. *Bioinformatics* 2009, **25**(14):1754-1760.

5. DePristo MA, Banks E, Poplin R, Garimella KV, Maguire JR, Hartl C, Philippakis AA, del Angel G, Rivas MA, Hanna M *et al*: **A framework for variation discovery and genotyping using next-generation DNA sequencing data**. *Nat Genet* 2011, **43**(5):491-498.

6. Newman AM, Bratman SV, Stehr H, Lee LJ, Liu CL, Diehn M, Alizadeh AA: **FACTERA: a practical method for the discovery of genomic rearrangements at breakpoint resolution**. *Bioinformatics* 2014, **30**(23):3390-3393.

7. Engelman JA, Mukohara T, Zejnullahu K, Lifshits E, Borras AM, Gale CM, Naumov GN, Yeap BY, Jarrell E, Sun J *et al*: **Allelic dilution obscures detection of a biologically significant resistance mutation in EGFR-amplified lung cancer**. *J Clin Invest* 2006, **116**(10):2695-2706.

8. Ercan D, Choi HG, Yun CH, Capelletti M, Xie T, Eck MJ, Gray NS, Janne PA: **EGFR Mutations and Resistance to Irreversible Pyrimidine-Based EGFR Inhibitors**. *Clin Cancer Res* 2015, **21**(17):3913-3923.

9. Nishino M, Suda K, Kobayashi Y, Ohara S, Fujino T, Koga T, Chiba M, Shimoji M, Tomizawa K, Takemoto T *et al*: **Effects of secondary EGFR mutations on resistance against upfront osimertinib in cells with EGFR-activating mutations in vitro**. *Lung Cancer* 2018, **126**:149-155.

10. Togashi Y, Sakamoto H, Hayashi H, Terashima M, de Velasco MA, Fujita Y, Kodera Y, Sakai K, Tomida S, Kitano M *et al*: **Homozygous deletion of the activin A receptor, type IB gene is associated with an aggressive cancer phenotype in pancreatic cancer**. *Mol Cancer* 2014, **13**:126.
