# Supplementary material for: A novel acquired EGFR-SEPT14 fusion confers differential drug resistance to EGFR inhibitors in lung adenocarcinoma
Source: Genes Dis. 2023 Apr 24;10(6):2241–4. doi: 10.1016/j.gendis.2023.02.019 (PMC10404941; doi:10.1016/j.gendis.2023.02.019)

Supplementary Figure-1

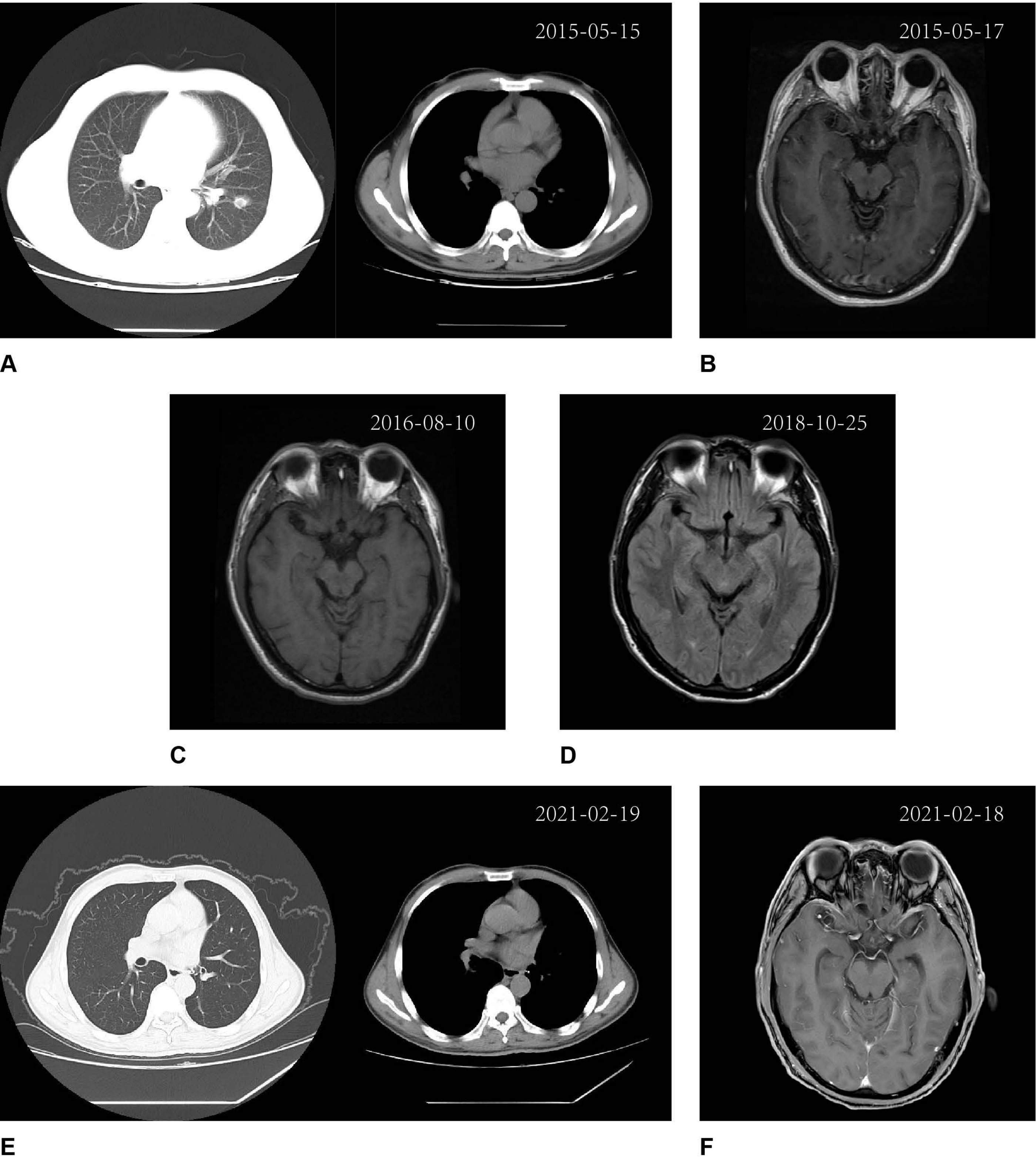

Supplementary Figure-2

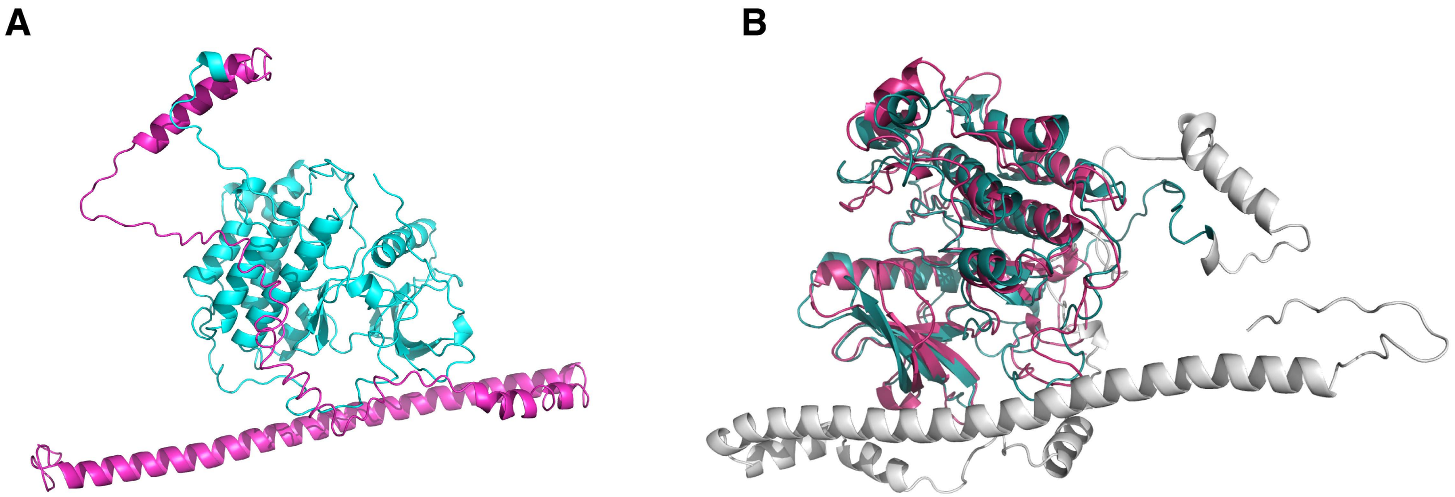

Supplementary Figure-3

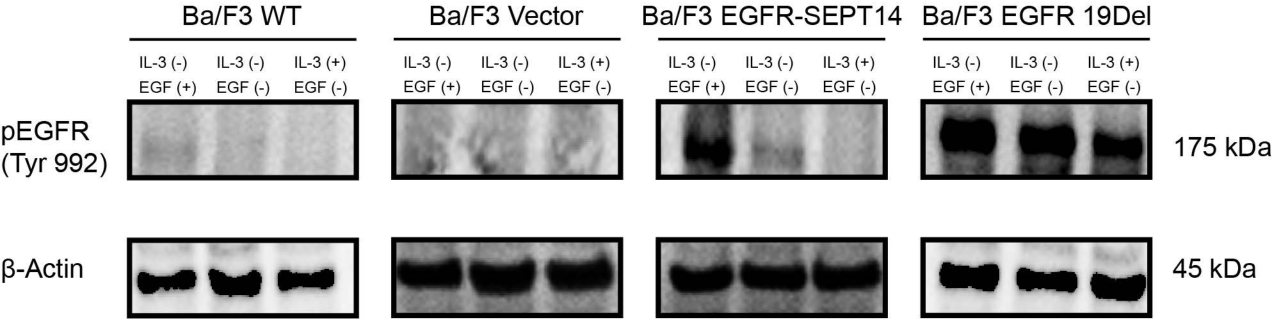

Supplementary Figure-4

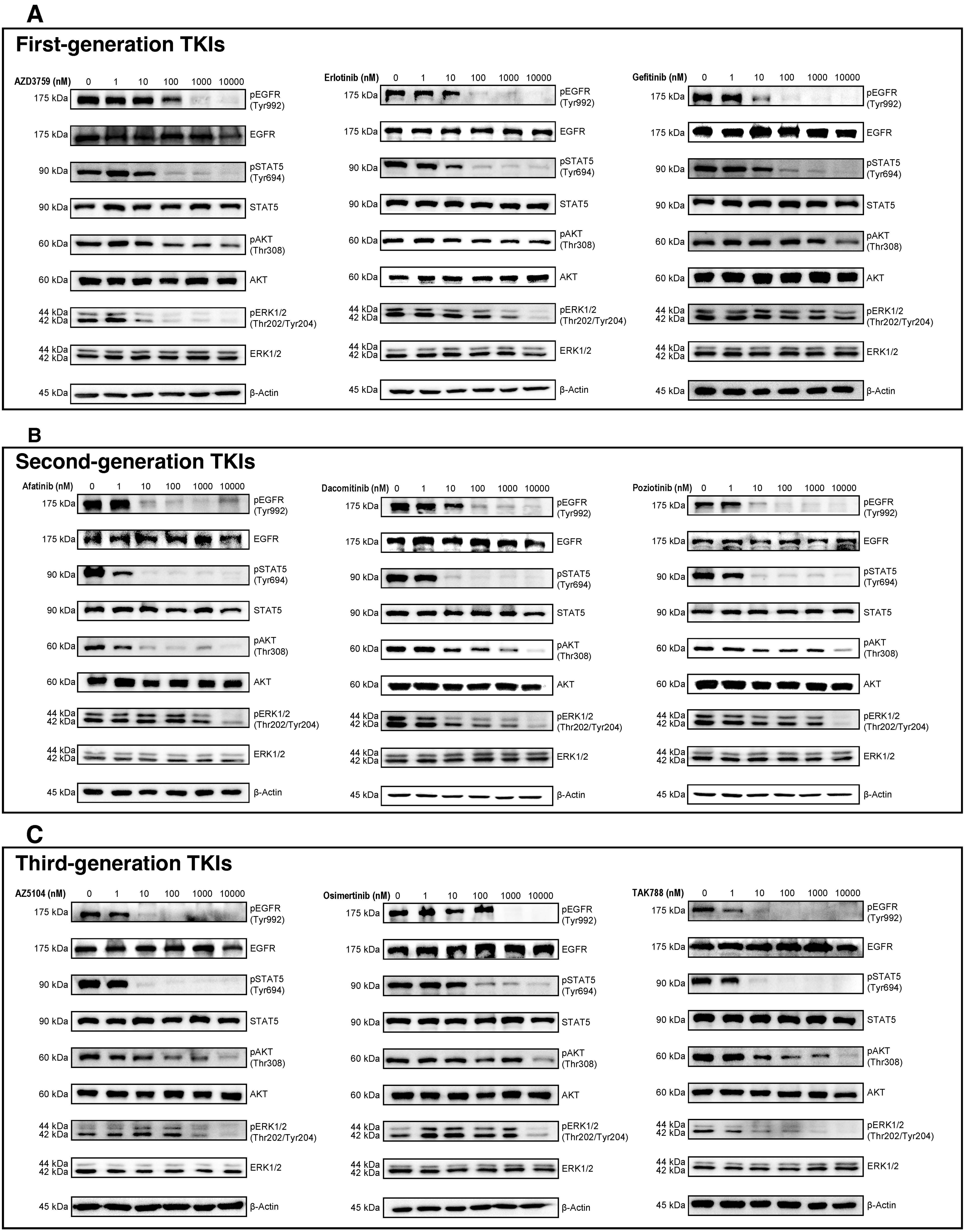

Supplement: Multimedia component 2 [file mmc2.pdf]
